# The use of silver diamine fluoride in a children's hospital: Critical analysis and action protocol

**DOI:** 10.1002/cre2.611

**Published:** 2022-07-22

**Authors:** Lluís Brunet‐Llobet, Beatriz Auría‐Martín, Yndira González‐Chópite, Pau Cahuana‐Bartra, Elias Isaack Mashala, Jaume Miranda‐Rius

**Affiliations:** ^1^ Department of Pediatric Dentistry, Hospital Sant Joan de Déu University of Barcelona Barcelona Spain; ^2^ Hospital Dentistry, Clinical Orthodontics and Periodontal Medicine Research Group (HDCORPEMrg) Institut de Recerca Sant Joan de Déu (IRSJD) Barcelona Spain; ^3^ Department of Odontostomatology, Faculty of Medicine and Health Sciences University of Barcelona Barcelona Spain; ^4^ Doctoral Programme in Medicine and Translational Research, Line of Odontostomatology. Faculty of Medicine and Health Sciences, Hospital Sant Joan de Déu University of Barcelona Barcelona Spain

**Keywords:** early childhood caries, patient with disability, silver diamine fluoride, special health care needs

## Abstract

**Objectives:**

The present critical analysis aims to propose an action protocol for the use of silver diamine fluoride (SDF) in pediatric patients in a hospital setting, especially for those who are currently awaiting hospital dental treatment under general anesthesia.

**Material and Methods:**

A literature search was performed in the PubMed/MEDLINE from 2009 to 2021 using the search terms “silver diamine fluoride”, “pediatrics silver diamine fluoride”, “application silver diamine fluoride”, and “AND” ‐ combined with terms: “potassium iodide”, “properties”, “adverse effects”, “early childhood caries”, “patient with disability”, “management”, “special health care needs patient”. Articles that recorded the type of teeth, application protocol, the concentration of the product, and possible complications of the treatment were selected.

**Results:**

Four hundred and sixteen related articles were obtained, of which 13 were finally chosen on the basis of the search criteria. The age at which the use of SDF was recommended ranged from the first year of life to 12 years, in most cases in primary teeth. The study populations varied in size from 53 to 799 patients. An analysis of the characteristics of SDF and its use in pediatric patients with dental caries was performed. The recommended concentration was 38% SDF, applied twice yearly. The main complication reported was staining. A decision algorithm was designed including SDF as an agent for caries control in patients attending the specific population of our hospital (divided into two groups: healthy children aged 0−4 years and patients with special health care needs (SHCNs) aged 0−18 years).

**Conclusions:**

SDF therapy appears to be effective in the control of caries in pediatric patients. We propose an action protocol for patients with early childhood caries to reduce risk, complications, and the progression of lesions. The protocol is aimed especially at pediatric patients who also have some systemic pathology, disability, SHCNs, and/or behavioral difficulties.

## 1 INTRODUCTION

Hospital dental care is essential in certain types of pediatric patients, either because of their special needs or because of the complexity of the interventions. Treating individuals with special health care needs (SHCNs) requires specialized knowledge, increased awareness and attention, and adaptive measures beyond what is generally considered routine (Velan & Sheller, 2013). Dental caries is a painful experience for children, especially in the advanced stages, and the consequences of untreated dental caries may be devastating. These consequences include hospitalizations and emergency room visits, high treatment costs, loss of school days, decreased ability to learn, and reduced oral health‐related quality of life (American Academy of Pediatric Dentistry, 2020a; Crystal et al., 2017).

The American Academy of Pediatric Dentistry (AAPD) defines early childhood caries (ECC) as the presence of one or more decayed primary teeth in a child under 6 years of age. The definition includes cavitated or noncavitated dental lesions, missing teeth due to caries, or filled tooth surfaces. ECC is considered severe when it affects children under 3 years of age (American Academy of Pediatric Dentistry, 2019a). ECC is one of the most prevalent health problems worldwide; it affects more than 500 million children and entails the risk of suffering multiple episodes of pain and oral infections, and may even affect permanent dentition (GBD 2015 Disease and Injury Incidence and Prevalent Collaborators, 2016; Kassebaum et al., 2017; Li & Wang, 2002; Saethre‐Sundli et al., 2020). Patient cooperation at these early ages may be poor, and the difficulty of managing these patients generates an increase in the demand for care at the hospital level (American Academy of Pediatric Dentistry, 2019b).

Pediatric patients with SHCNs frequently present dental caries and gingivitis, mainly due to the difficulty of maintaining correct oral hygiene. Other problems such as oral breathing, dental malocclusion, cariogenic diet, and secondary effects due to the medication administered can play an important role in the appearance of caries (Pini et al., 2016). Some authors report high rates of caries in 75% of patients with SHCNs (Lazzaretti et al., 2013).

In pediatric dentistry, the treatment and management of dental caries in precooperative children and children with SHCNs is a challenge. Due to poor patient cooperation, sedation or general anesthesia (GA) is sometimes needed (Coté & Wilson, 2016; Crystal et al., 2017). However, in 2016, the United States Food and Drug Administration (FDA) warned of possible impaired brain development in children under 3 following exposure to certain agents used in GA. The FDA recommended that healthcare providers should carefully consider the necessity of appropriate anesthesia in children of this age, especially for procedures that may take more than 3 h or if multiple procedures are planned in children under the age of 3 (Olutoye et al., 2018). Moreover, the limited availability of operating rooms for performing dental treatment under GA or deep sedation means that these patients may face long waiting lists (Jamieson & Roberts‐Thomson, 2006). In this scenario, there is a need for alternative treatments that can control the progress of dental caries, a highly destructive condition that evolves rapidly in pediatric patients and in patients with SHCNs (Oliveira et al., 2019).

Traditionally, fluorides have been widely used for the control and prevention of dental caries. Despite their clear benefits in enamel remineralization, high‐dose fluoride intake can be harmful. In certain geographical areas where public drinking water naturally has high fluoride concentrations, high rates of dental and even skeletal fluorosis have been found (Miranda‐Rius et al., 2020).

**Figure 1:**
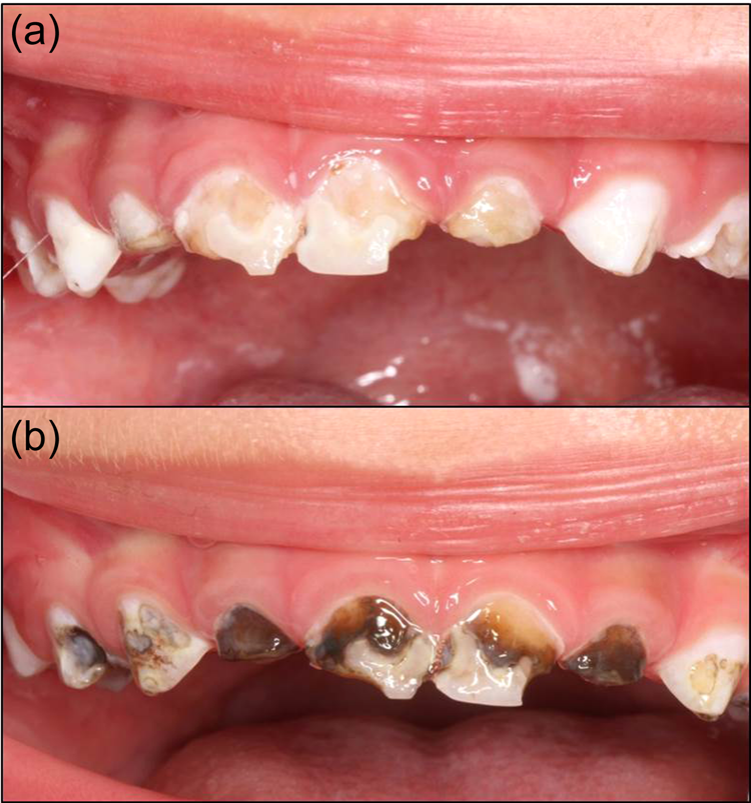
Clinical image (a) before, and (b) after using SDF. Note its dark color after the aplication of SDF. SDF, silver diamine fluoride.

In the field of preventive dentistry, the benefits of topical fluoride at adequate doses far outweigh the drawbacks. Silver diamine fluoride (SDF) is a dental medication for topical use, formulated in liquid form at 38%, with a pH of 10, and comprising 24.4%−28.8% volume of silver, 5.0%−5.9% fluoride, 8% ammonia, and 62% water. SDF inhibits the degradation of dentin collagen and presents anticariogenic, remineralizing, and bactericidal properties that are particularly useful in pediatric or SHCNs (Crystal et al., 2017; Rosenblatt et al., 2009; Zhao et al., 2018). Currently, certain formulations also incorporate potassium iodide, which enhances the antimicrobial effect and also seems to reverse or minimize the dark coloration that SDF produces on carious teeth after its application (Haiat et al., 2021; Roberts et al., 2020) (Figure 1).

SDF was introduced in Japan in the 1960s for dental use and was later approved by the FDA in 2014 (Horst, 2018; Yamaga et al., 1972). It was initially described as a desensitizing agent but at the same time it was used off‐label for caries control. Finally, in 2021, the WHO declared SDF an essential medicine for dental health, along with fluoride toothpaste and glass ionomer (Seifo et al., 2020; World Health Organization, 2021). Currently, SDF is regarded as an affordable, easy‐to‐apply product with proven efficacy (Gao et al., 2021; Horst & Heima, 2019). It is emerging as an agent for daily use in our hospital environment, where we attend a large pediatric population currently requiring dental treatment under GA or deep sedation (Wright & White, 2017).

The use of SDF combined with the Atraumatic Restorative Technique (ART), termed Silver Modified Atraumatic Restorative Technique (SMART), has also been recommended (Alvear et al., 2016). In this process, the caries is arrested and the tooth restored using glass ionomer (Duangthip et al., 2017).

The main aim of this study is to describe the characteristics of SDF and indicate its current clinical applications in a hospital with universal coverage in healthy children with ECC aged between 0 and 4 years and in pediatric patients aged 0−18 years with SHCNs. We also present an algorithm for the incorporation of SDF into dental care at a referral hospital.

## 2 METHODS

A bibliography search was carried out in the PubMed/MEDLINE database for articles published in the period 2009−2021. The primary aim of this critical review was to identify all original articles and literature reviews describing treatment with SDF and which included the following data: (a) type of teeth, (b) the application protocol, (c) product concentration, and (d) possible complications.

The following keywords were used in the search: “silver diamine fluoride” (MeSH) (*n* = 416), “pediatrics silver diamine fluoride” (MeSH) (*n* = 152), “application silver diamine fluoride” (MeSH) (*n* = 657), and “AND”—combined with terms: “potassium iodide”, “properties”, “adverse effects”, “early childhood caries”, “patient with disability”, “management”, “special health care needs patient”.

The selection of eligible studies was carried out in three stages: (1) assessment of titles; (2) review of abstracts, and (3) elimination of duplicate articles. Case series, guidelines, letters to editors, and studies of participants with follow‐up were included.

## 3 STUDY SELECTION AND RESULTS

Publications by scientific dentistry societies were included, such as the AAPD Clinical practice guideline (Crystal et al., 2017) and reviews in journals published by dental associations such as the American Dental Association (ADA) (Zhao et al., 2018).

Thirteen articles were eventually selected to assess the characteristics, advantages/disadvantages, uses, and efficacy of SDF in pediatric patients (Table 1). Eleven of the publications selected centered on primary dentition (Abdellatif et al., 2020; Dos Santos et al., 2012; Duangthip et al., 2016; Fung et al., 2018; Gao et al., 2020; Jiang, Wong, et al., 2020; Mabangkhru et al., 2020; Turton et al., 2021; Vollú et al., 2019; Yee et al., 2009; Zhi et al., 2012) and two clinical trials on first permanent molars (Liu et al., 2012; Monse et al., 2012). All the publications studied the effect of SDF in carious lesions in dentin except Liu et al. (2012), whose study focused on enamel. As regards the most efficacious and effective way to use SDF, the studies selected have shown that an SDF concentration of at least 30% is effective for arresting carious lesions in enamel and/or dentin (Fung et al., 2018; Liu et al., 2012; Monse et al., 2012; Yee et al., 2009). However, if we compare SDF with different preventive products or techniques, its superiority has not been demonstrated (Abdellatif et al., 2021; Dos Santos et al., 2012; Duangthip et al., 2016; Gao et al., 2020; Jiang, Mei, et al., 2020; Liu et al., 2012; Mabangkhru et al., 2020; Monse et al., 2012; Turton et al., 2021; Vollú et al., 2019; Zhi et al., 2012). The information on SDF as an agent that stops or slows the progression of dental caries was classified according to children's age, type of dentition, number of patients in the study, treatment protocol, follow‐up, duration of study, and results or conclusions of the paper.

**Table 1:** List of studies with protocols for the application, treatment, and follow‐up of SDF.

Author and year of study	Age of children examined (years)	Type of dentition/Site of caries	Patients (*n*)	Treatment protocol. Group/no. applications/material/(*t*)	Follow‐up protocol (months)	Duration of study (months)	Results and conclusions
Turton et al. (2021)	3−12	– Primary dentition. – Lesion in dentin.	261	Group 1:1/SDF 38%/6 months. Group 2:1/SDF 38% + KI/6 months. Group 3:1/AgF/6 months. Group 4:1/AgF + KI/6 months.	Every 6 months.	12	The effectiveness of 38% SDF and AgF is similar but decreases if KI is applied.
Abdellatif et al. (2021)	3−8	– Primary dentition. – Lesion in dentin.	53	Atraumatic removal with ART. Group 1: 1/SDF 38%/6 months. Group 2: 1/GIC (ART)/single.	Every 6 months.	12	Similar effectiveness with SDF at 38% and ART.
Jiang, Wong, et al. (2020)	3−4	– Primary dentition. – Lesion in dentin.	172	Atraumatic removal with ART. Group 1:1/SDF 38% + GIC (ART)^a^/single. Group 2:1/Placebo + GIC (ART)^a^/single.	Every 6 months.	24	The application of SDF at 38% does not increase the effectiveness of ART.
Gao et al. (2020)	3−4	– Primary dentition. – Lesion in dentin.	345	Group 1: 1/AgNO_3_ 25% + FV 5%/6 months. Group 2:1/SDF 38% + placebo varnish/6 months.	Every 6 months.	30	Same effectiveness with both techniques.
Mabangkhru et al. (2020)	1−3	– Primary dentition. – Lesion in dentin.	263	Group 1: 1/SDF 38%/6 months. Group 2: 1/FV 5%/6 months.	Every 6 months.	12	Higher effectiveness with SDF at 38% versus FV at 5%.
Vollú et al. (2019)	2–5	– Primary dentition. – Lesion in dentin.	56	Atraumatic removal with ART. Group 1: 1/SDF 30%/single. Group 2: 1/GIC (ART)/single.	At 3, 6, and 12 months.	12	The effects of SDF and ART are similar.
Fung et al. (2018)	3−4	– Primary dentition. – Lesion in dentin.	799	Group 1: 1/SDF 12%/12 months. Group 2: 1/SDF 12%/6 months. Group 3: 1/SDF 38%/12 months. Group 4: 1/SDF 38%/6 months.	Every 6 months.	30	The application of SDF at 38% is more effective than SDF at 12%: it can be applied more frequently in children with poor oral hygiene.
Duangthip et al. (2016)	3−4	– Primary dentition. – Lesion in dentin.	275	Group 1: 1/SDF 30%/12 months. Group 2: 3/SDF 30%/21 days^b^. Group 3: 3/FV 5%/21 days^b^.	Every 6 months.	18	The application of SDF at 30% is the most effective therapy regardless of the frequency and mode of application.
Dos Santos et al. (2012)	5−6	– Primary dentition. – Lesion in dentin.	91	Atraumatic removal. Group 1: 1/GIC (IRT)/single. Group 2: 1/SDF 30%/single.	Every 6 months.	12	The application of SDF at 30% was more effective than the technique with GIC.
Liu et al. (2012)	Media: 9.1	– First permanent molars. – Lesion in enamel.	485	Group 1: 1/SDF 38%/12 months. Group 2: 1/FV 5%/6 months. Group 3: 1/Resin sealant/single. Group 4: Placebo.	Every 6 months.	24	All treatments studied are effective compared to placebo.
Monse et al. (2012)	6−8	– First permanent molars. – Lesion in dentin.	704	Group 1: 1/SDF 38%/single. Group 2: 1/GIC (ART)/single. Group 3: Control.	Not well defined.	18	Treatment with ART was more effective than treatment with SDF at 30%.
Zhi et al. (2012)	3−4	– Primary dentition. – Lesion in dentin.	181	Atraumatic removal. Group 1: 1/SDF 38%/12 months. Group 2: 1/SDF 38%/6 months. Group 3: 1/GIC/12 months.	Every 6 months.	24	There were no differences in lesion arrest with SDF or GIC. SDF at 38%/6 months reduces the caries process.
Yee et al. (2009)	3−9	– Primary dentition. – Lesion in dentin.	634	Group 1: 1/SDF 38%/single. Group 2: 1/SDF 38% + tannic acid/single. Group 3: 1/SDF 12%/single. Group 4: Control.	At 6, 12, and 24 months.	24	The application of SDF at 38% is more effective than SDF at 12% or the association with other components.

Abbreviations: AgF, silver fluoride solution; AgNO_3_, silver nitrate; ART, atraumatic restorative treatment; FV, varnish with sodium fluoride; GIC, glass ionomer cement; IRT, interim restorative treatment; KI, potassium iodide; SDF, silver diamine fluoride.

^a^ Treatment with ART 10 days after the application of SDF.

^b^ One weekly application for 3 weeks.

**Figure 2:**
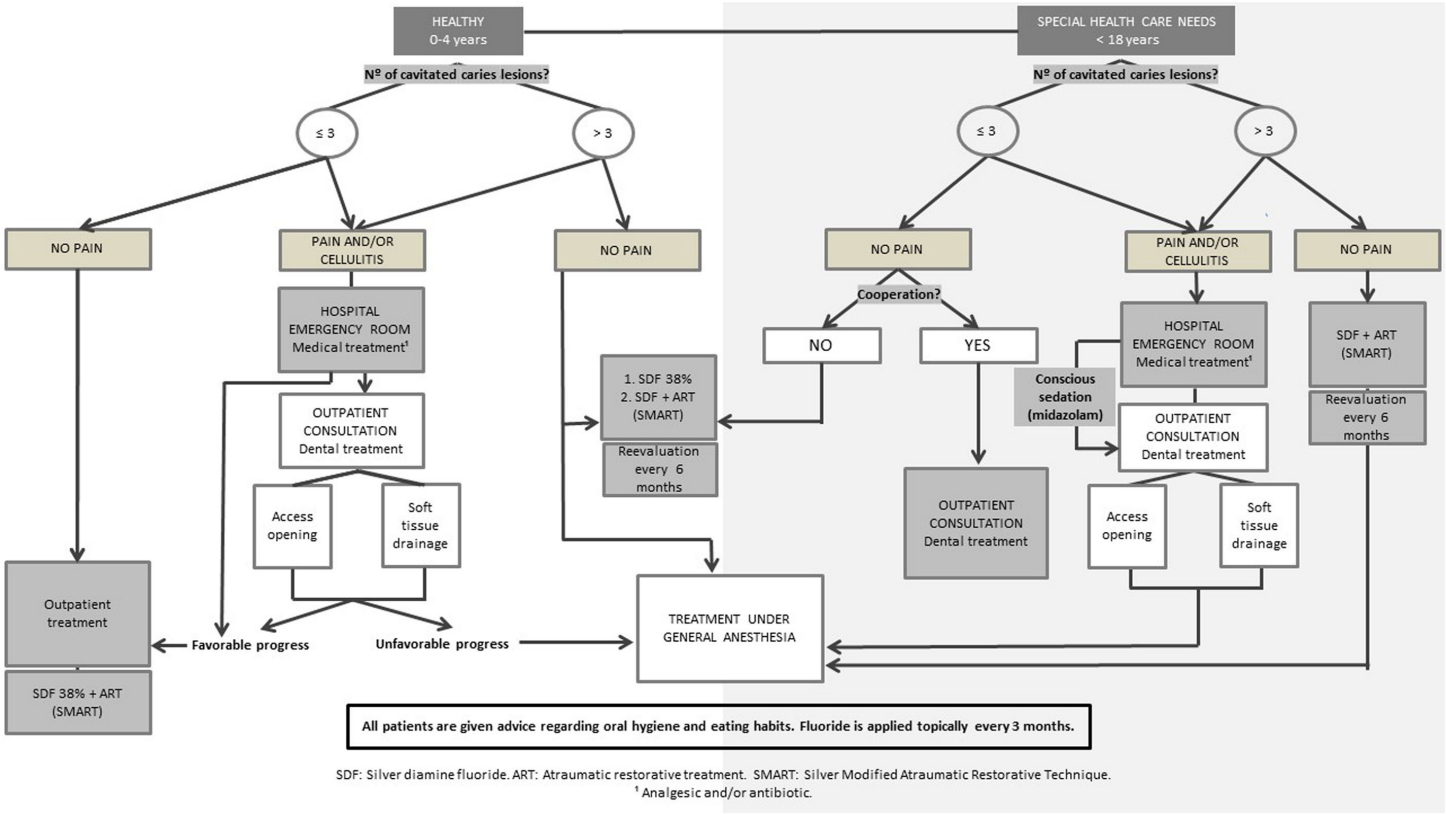
Algorithm for dental treatment at a hospital pediatric dentistry service inside the public health system with universal coverage.

Here we discuss the advances in the formulations of SDF and present a flow chart depicting the incorporation of this drug in the clinical decision‐making process (Figure 2).

## 4 DISCUSSION

Our hospital pediatric dentistry department attends to an average of 8600 visits/year, of which 580 patients receive dental treatment under general anesthesia for multiple cavities and 260 receive oral surgery. In pediatric patients with multiple caries, conventional restorative treatment may be difficult to perform. This is particularly the case in very young children, those with SHCNs, and those who are medically compromised (Crystal & Niederman, 2016). In order to overcome these difficulties dentists are obliged to apply advanced behavior management techniques, including sedation and GA. These practices increase the costs of the procedure and carry certain intrinsic risks for the patient (Horst, 2018). Furthermore, the postponement of routine dental treatment during the COVID‐19 pandemic has inevitably created a considerable backlog of children with untreated caries. In this context, the use of nonaerosol‐generating minimally invasive procedures in clinical dental care is increasing. For all these reasons, there is a clear need to find effective, safe, and accessible alternatives for the treatment of caries lesions in these groups of patients (Crystal & Niederman, 2019; Gao et al., 2021; Mendiratta et al., 2021).

SDF is a product that combines the antibacterial effects of silver with the remineralizing effects of fluoride, and it is considered a very useful therapeutic agent for controlling carious lesions. Multiple in vitro studies have demonstrated its efficacy for reducing specific cariogenic bacteria (Mei, Li, et al., 2013; Piovesan et al., 2021) and also its remineralizing potential on enamel and dentin (Chu & Lo, 2008; Mei, Ito, et al., 2013; Yu et al., 2018).

In 2017, the American Academy of Pediatric Dentistry (AAPD) published a set of guidelines, which intended to incorporate this treatment for caries arrest in pediatric patients (Crystal et al., 2017).

A major advantage of this simple, easy, and quick‐to‐apply product is that it can be used as a desensitization technique in difficult‐to‐manage patients. The fact that SDF treatment is noninvasive increases their confidence in their dentist (Hu et al., 2020). In addition, in young patients, halting the lesions allows us to gain time for the patient to mature and be able to cooperate more in conventional dental treatment (Wright & White, 2017).

The search for innovative approaches to treat dental caries has led to the publication of clinical trials to verify the efficacy and therapeutic action of SDF (Dos Santos et al., 2012; Monse et al., 2012; Yee et al., 2009), its effectiveness at different concentrations (Fung et al., 2018; Yee et al., 2009) and its ideal frequency of application (Duangthip et al., 2016; Fung et al., 2018; Zhi et al., 2012). The results of these studies have shown that an SDF concentration of 38% is effective for arresting carious lesions in enamel and/or dentin.

The American Dental Association (ADA) and the AAPD provide evidence‐based guidelines for the nonrestorative treatment of carious lesions. Both recommend biannual applications of SDF at a concentration of 38% to stop advanced cavitated lesions in primary teeth without pulpal involvement, but stress that additional applications may occasionally be necessary (Mei, Li, et al., 2013).

SDF has also been studied and compared with other preventive agents such as fluoride varnish (Duangthip et al., 2016; Liu et al., 2012; Mabangkhru et al., 2020), resinous pit/fissure sealant in enamel caries lesions (Liu et al., 2012), silver fluoride solution (Turton et al., 2021), and silver nitrate (Gao et al., 2020). However, there is no consensus regarding the superiority of SDF over other products.

Comparisons of the arrest of caries lesions with the application of SDF or the use of atraumatic restorative treatment (ART) have not found significant differences between the two techniques in terms of effectiveness (Abdellatif et al., 2021; Dos Santos et al., 2012; Monse et al., 2012; Vollú et al., 2019; Zhi et al., 2012). However, Monse et al. (2012) concluded that a single application of 38% SDF did not prevent dentin caries lesions, while ART was able to do so. For their part, Jiang, Wong, et al. (2020) investigated the effectiveness of the ART technique with or without previous application of SDF at 38% and concluded that prior application of this agent did not increase the effectiveness of ART.

As regards the need to remove the affected dentin before the application of SDF, the results are inconclusive. Indeed, not all studies use the same methodology: some authors analyze the effectiveness of SDF applied directly on the caries (Duangthip et al., 2016; Fung et al., 2018; Gao et al., 2020; Mabangkhru et al., 2020; Turton et al., 2021; Yee et al., 2009), while others evaluate its efficacy after partial removal of the lesion (Dos Santos et al., 2012; Zhi et al., 2012). To our knowledge, no comparative studies of the two techniques (i.e., removal vs. nonremoval before SDF application) have been published.

The current interest in SDF has led to the publication of several systematic reviews describing its ability to arrest or prevent carious lesions (Contreras et al., 2017; Crystal & Niederman, 2016; Jabin et al., 2020; Seifo et al., 2019). Like the present study, these reviews compare its efficacy and effectiveness with other treatments such as ART and fluoride varnish. They report the use of SDF in both the primary and permanent dentition of children.

As for side effects, the reviews or trials do not report any acute adverse reactions with SDF, although minor undesirable effects such as transient gingival irritation and metallic taste have been described in a small number of patients (Turton et al., 2021). A study in adults found that a very small number of patients developed mild gingival erythema within 24 h of SDF application, but that after 7 days no differences were found with respect to baseline (Castillo et al., 2011). Other authors studying the adverse effects of SDF in children found that 6.6% of parents reported dental and gum discomfort 7 days after its application, while gum swelling and gum bleaching were reported in 2.8% and 4.7% of cases respectively (Duangthip et al., 2018).

Although no acute toxicity has been reported with SDF used at therapeutic doses, the high concentration of fluoride and silver (a heavy metal) have raised concern, especially when repeated applications are necessary for very young children (Gotjamanos, 1997). In a study on adults, Vasquez et al. (2012) described the pharmacokinetics of SDF after oral application, concluding that serum concentrations of fluoride and silver demonstrated a low risk of toxicity when used only occasionally in this population. In a study on rats, Chen et al. (2020) suggested that the conventional application of SDF on the teeth to prevent or stop dental caries results in silver concentrations in plasma and tissue that were far below toxic levels.

The main side effect of SDF treatment is the dark staining of the carious dental tissue, which has raised concerns among parents (Vollú et al., 2019). However, Duangthip et al. (2018) found that parents' satisfaction with their children's dental appearance after 30 months was 71%, and Mabangkhru et al. (2020) concluded that SDF does not negatively impact parental satisfaction regarding the esthetic appearance of their children's teeth, especially in patients with SHCNs (Almarwan et al., 2021).

Certain products, including potassium iodide, have been proposed for minimizing the dark discoloration of the tooth after the application of SDF (Nelson et al., 2016). However, in a study on adults, Li et al. (2016) reported that the subsequent application of potassium iodide had no effect in reducing the blackish staining of root caries, especially in the long term, and, in 2021, Turton et al. reported that SDF was less effective when combined with potassium iodide (Turton et al., 2021).

Another disadvantage of SDF is that it does not have the ability to restore teeth affected by caries. In a study of its effect on the bond strength of adhesives on the dental surface, Siqueira et al. (2020) concluded that SDF did not alter their degree of chemical conversion or their microtensile bond strength. In addition, in a recent review, Jiang, Mei, et al. (2020) were also unable to conclude that the application of SDF influenced the bond strength of adhesive restorative materials or glass ionomer cements, due to the heterogeneity of the studies included. As a consequence, SMART technique has been proposed (Alvear et al., 2016). This simple, conservative technique aims to stop caries lesions by applying SDF, and then (either in the same visit or later) by restoring the tooth with a glass ionomer cement. However, despite its obvious advantages, authors such as Jiang, Wong, et al. (2020) found no significant differences when comparing SMART with the conventional ART technique.

Finally, we present the decision‐making diagram used in this public hospital setting for the implementation of SDF, taking into account the patient's age, medical situation, and the number of dental caries. This algorithm also describes our therapy in patients aged 0−4 years who come to the emergency room for acute pain or extensive cellulitis who undergo medical, pharmacological, and/or dental treatment.

Healthy children aged 0−4 years with ≤3 carious lesions are not candidates for treatment at our hospital, since the agreement in place stipulates that healthy infants under 4 years old with this number of lesions, can be treated by conventional behavioral guidance techniques at outpatient centers and do not require treatment under GA. In fact, as indicated by the AAPD, “the use of general anesthesia is contraindicated for healthy, cooperative patients with minimal dental needs.” This is because the risk−benefit ratio does not justify the use of techniques such as GA in patients with a low number of caries lesions, except in the case of dental pain (American Academy of Pediatric Dentistry, 2020b).

In children under 4 years of age with poor cooperation and up to three lesions, in our context, pediatric dentists can use advanced nonpharmacological techniques such as protective stabilization, as described by the AAPD. This procedure is indicated in patients who cannot cooperate due to their lack of maturity or mental or physical disability (Clinical Affairs Committee—Behavior Management Subcommittee, 2015).

The use of products such as SDF for the arrest of caries lesions seems to be well accepted by the parents of young patients and is regarded as preferable to the use of GA (Crystal et al., 2017). For this reason, in these cases, our algorithm proposes the use of SDF and/or ART at outpatient centers since these treatments have been described for the management of patients with ECC or SHCNs in the dental office (Mabangkhru et al., 2020).

In our decision‐making protocol, healthy patients aged 0−4 years with four carious lesions or more who do not currently present pain are scheduled for treatment under GA, as established in the existing agreement between our hospital and the regional government's public health service. Due to the long waiting lists at our center, in these cases, the possibility of preventing the progression of caries lesions in patients by using the 38% SDF or SMART is considered, with reassessment of the child every 6 months until the definitive surgery (Wright & White, 2017).

In the case of patients with SHCNs aged 0−18 years who present <3 caries lesions, if there is no pain, the choice of treatment will depend on the patient's ability to cooperate. In uncooperative children, we opt for simpler strategies such as SDF or SMART (Crystal et al., 2017; Mendiratta et al., 2021), while in those able to cooperate, the conventional treatment indicated in each case will be carried out at the hospital's outpatient clinics. Patients in this same group who present four or more carious lesions without painful symptoms undergo comprehensive treatment under GA. Initially, SDF and/or ART are applied to stop the progression of the caries lesions until the time of the definitive treatment in the operating room.

Due to their age, underlying pathology or number of carious lesions, the patients we attend at our pediatric hospital are considered “high risk” according to the AAPD's Best Practices: Caries‐Risk Assessment and Management for Infants, Children, and Adolescents. We therefore consider dietary advice to be essential in all cases as well as instructions in oral hygiene techniques, accompanied by quarterly fluoridations until this risk has decreased (American Academy of Pediatric Dentistry, 2020c).

Our critical analysis has some limitations that should be mentioned. Clearly, it is very focused on the two groups of patients that we see daily at our Maternal and Child University Hospital (healthy patients aged 0−4 years and patients with SHCNs aged 0−18 years). Due to these special circumstances inherent to our center, the algorithm that we describe only applies to the introduction of SDF in the hospital setting for this type of pediatric patients.

All in all, the management of ECC and of carious lesions in patients with SHCNs represents a major challenge for the dental clinician. Currently, SDF is a useful tool in the control of this pathology, either as a definitive treatment or as a remineralizing agent that minimizes the progression of caries and avoids complications. It may also help to reduce the long waiting lists for treating these patients under GA, given the limited availability of operating rooms.

## 5 CONCLUSIONS

- SDF is a useful therapeutic strategy to prevent and control the carious lesions in pediatric patients.
- The disadvantage of SDF is the dark stains that appear in the treated areas and the impossibility of restoring the destroyed dental tissue.
- The algorithm we describe for the care of young patients with multiple caries lesions or children with SHCNs may be helpful for decision‐making in the hospital setting.

## AUTHOR CONTRIBUTIONS

Lluís Brunet‐Llobet, Beatriz Auría‐Martín, Yndira González‐Chópite, Elias Isaack Mashala, and Pau Cahuana‐Bartra carried out the literature search and selection of manuscripts for review. Lluís Brunet‐Llobet, Beatriz Auría‐Martín, Yndira González‐Chópite, and Jaume Miranda‐Rius drafted the article. All the authors critically revised the manuscript for important intellectual content. All the authors have read and approved the final manuscript.

## CONFLICT OF INTEREST

The authors declare no conflict of interest.

## Data Availability

Data sharing not applicable to this article as no data sets were generated or analyzed during the current study.
